# Deaths in New York

**Published:** 1858-03

**Authors:** 


### DEATHS IN NEW YORK.

| | 1853. | 1854. | 1855. | .856. | 1857. |
|---|---|---|---|---|---|
| From all causes, | 22,702 | 28,568 | 22,787 | 21,658 | 23,333 |
| Throat and Lung Diseases, | 5,648 | 6,174 | 5,770 | 5,382 | 5,418 |
| Consumption, only, | 2,739 | 3,032 | 2,624 | 2,478 | 2,814 |

ONE year ago, it was claimed by a city print, and blindly copied over the country, that the practice of medicated inhalation in consumption had been so successful, that the mortality from that disease had diminished twenty-five per cent. This statement led multitudes to "try" the treatment. "Inhalation" offices were opened in all directions. The newspapers gave column after column, and whole pages at a time, at an expense of five hundred dollars a page, to the praise of this *new* mode of cure. Intelligent editors indorsed the fallacy in pointed editorials; the "reasonableness of the thing" was on all tongues. It was claimed that the "Discoverers" of this new mode of practice would, in a short time, by their accumulated observations and increasing skill in administration, still farther reduce the mortality. A year has passed, and Time, the great truth-teller, reveals a different story. In 1856, there were fewer deaths from consumption, by *one hundred and forty-six*, but in 1857 there were *more* deaths from that disease than in 1856, by *three hundred and thirty six*, so that the "increased skill" seems to have increased the mortality.

It is for the editors of New York to say how they shall atone to their subscribers for the agency they had in recommending to them a cure for a disease, which in reality, as time and figures tell, has no efficiency whatever, in the special direction claimed.

*More* than one half of all the deaths in New York, during 1857, were of children under three years of age!

Twenty-three per cent, or nearly one-fourth of all who died, perished from some form of diseases affecting the throat and lungs.

All these diseases of the throat and lungs are brought on by habits of life, which owe themselves to our ignorance, or our recklessness, and consequently are avoidable diseases; and this fearful scourge will prevail increasingly, until we make it a part of the education of every child HOW TO TAKE CARE OF THE HEALTH. Twelve per cent, or one out of every eight, died of consumption in New York city, during 1857.

Twice as many colored persons died of consumption as whites, according to the total number of deaths from each class.

We are indebted to the ready courtesy of GEORGE F. FALLS, M.D., the Secretary of the City Inspector's Department, for the figures used; and none who know him will doubt their correctness. Inspector MORTON has shown his sagacity in selecting one who fills his laborious office so well.

